# Noninvasive sampling of the distal airspace via HME-filter fluid is not useful to detect SARS-CoV-2 in intubated patients

**DOI:** 10.1186/s13054-021-03549-x

**Published:** 2021-03-30

**Authors:** Joerg Reifart, Christoph Liebetrau, Christian Troidl, Katharina Madlener, Andreas Rolf

**Affiliations:** 1 Department of Cardiology, Kerckhoff Heart Center, Benekestr. 2-8, 61231 Bad Nauheim, Germany; 2 DZHK (German Center for Cardiovascular Research), Partner Site RheinMain, Frankfurt am Main, Germany; 3 Department of Haemostaseology and Transfusion Medicine, Kerckhoff-Klinik, Bad Nauheim, Germany

**Keywords:** SARS-CoV-2, PCR, BAL, HME, Infection

Sampling for SARS-CoV-2 is often carried out via bronchoscopy in intubated patients if upper respiratory samples at nasopharyngeal and oropharyngeal sites are negative or cannot be readily obtained [1]. PCR for detection of SARS-CoV-2 has been reported to have the highest sensitivity in lower respiratory tract samples [2]. Because bronchoscopy is an aerosol-generating procedure, alternative sampling methods with a similar sensitivity are of value. Previously, it has been demonstrated that heat moisture exchanger (HME) filter fluid had a composition approximating that of the fluid in the distal airspace (which is obtained during bronchoalveolar lavage) and can be used to test for pathogens [3–5].

The present investigation examined whether performing PCR for SARS-CoV-2 on unprocessed HME filter fluid is a viable method to test patients for infection instead of performing bronchoalveolar lavage via bronchoscopy.

Patients who had tested positive for SARS-CoV-2 and were treated with invasive ventilation for at least 12 h were enrolled prospectively (Fig. 1). The enrollment period was from May 2020 to December 2020. The initial test yielding the positive result was conducted using a nasopharyngeal swab.

**Fig. 1:**
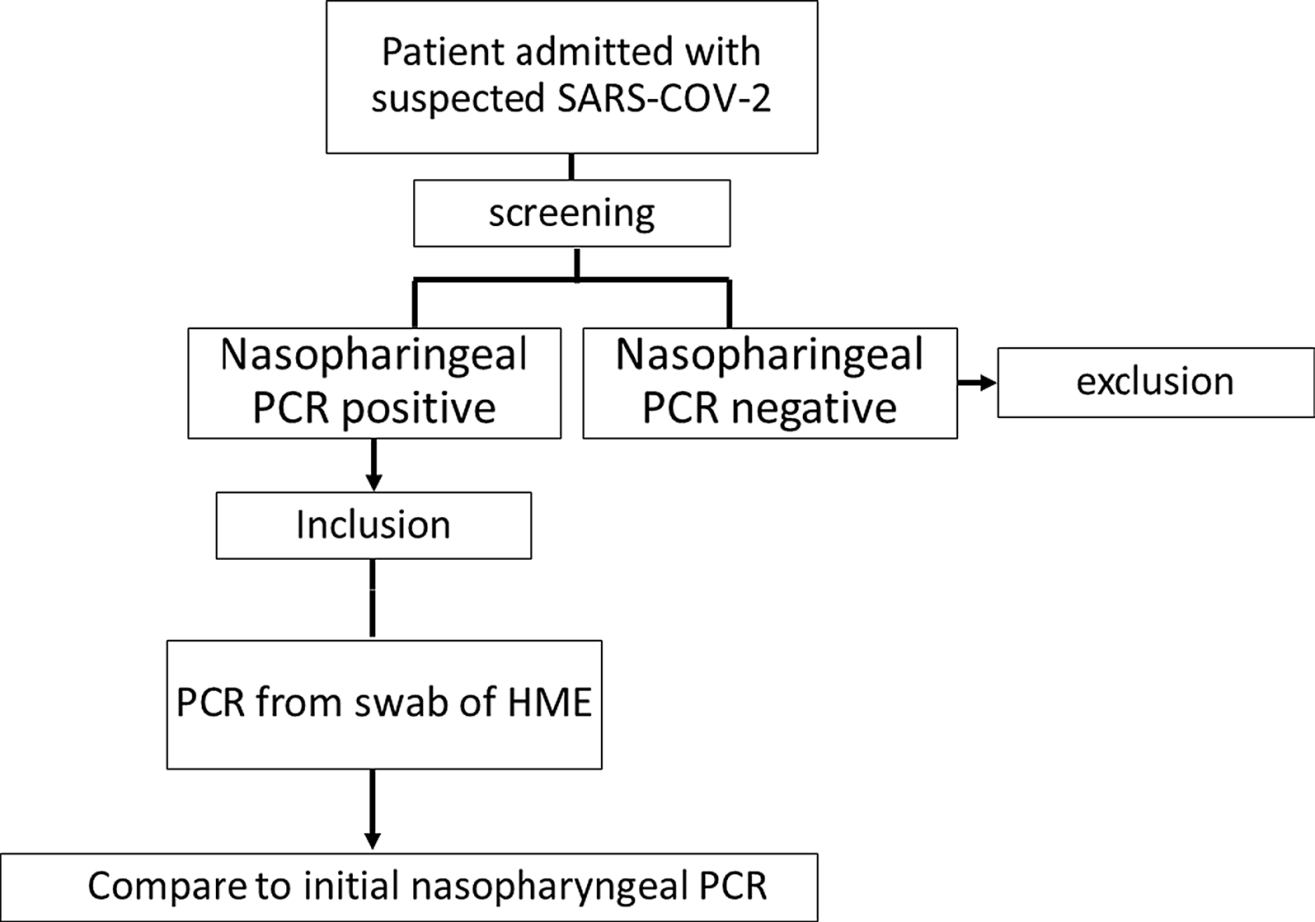
Flowchart of study design

HME filters were swabbed with a regular swab on the septic side of the filter. The swabs were then placed in virus medium. SARS-CoV-2 PCR test systems used were either the BD MAX™ System with BioGX SARS-CoV-2 reagents (BD Life Sciences, Sparks, Maryland, USA) or the Hain Lifescience FluoroType® SARS-CoV-2 plus (Hain Lifescience GmbH, Nehren, Germany). Results of the tests of samples from the HME filters were compared with the results from the nasopharyngeal swab.

A sample size of 60 patients appeared to be adequate to compare the two different test methods. This report provides data for the initial 4 patients included in this prospective population.

Informed consent was acquired. If the patient was not able to provide informed consent, inclusion occured via an investigator consilium ("Giessener Lösung"). The ethics board of the state of Hessen, Germany, approved the study (AZ 79/20). The data collected from all patients were pseudonymized and entered into a database.

The trial was retrospectively registered on November 1st 2020 (DRKS registry; registration: DRKS00023494).

Four patients with positive nasopharyngeal swabs were enrolled in the study before an interim analysis of the results showed that it was unlikely that a sensitivity of greater than 90% would be reached. The enrollment was stopped prematurely. Of the 4 HME filter samples assayed, 3 did not test positive for SARS-CoV-2. All patients still tested positive in a bronchoalveolar lavage sample at a later point in time. Further results are provided in Table 1.

**Table 1:** Patient and testing data

Variable	Patient 1	Patient 2	Patient 3	Patient 4
Age, y	72	85	79	66
Female sex	Yes	No	No	Yes
Body-mass index, kg/m [2]	33	27	24	33
CT/chest X-ray morphology for COVID-19	Yes	Yes	Yes	Yes
Time from first positive PCR test until HME swab, h	74	19	222	235
Time on ventilator until HME swab, h	68	19	212	159
Time on ventilator (total), h	494	243	369	159
SARS-CoV-2-positive HME PCR samples	Yes	No	No	No
SARS-CoV-2-positive PCR samples after HME PCR testing (BAL)	Yes	Yes	Yes	Yes
Therapy with corticosteroid	Yes	Yes	Yes	Yes
Therapy with azithromycin	Yes	Yes	Yes	Yes
Antiviral medication	No	No	No	No
Death within 30 days	No	Yes	Yes	No

Bronchoscopy with bronchoalveolar lavage is commonly used to test for SARS-CoV-2 in intubated patients. This study was designed to determine whether sampling HME filter fluid may present a feasible and safer alternative. Our preliminary data showed that HME filter fluid alone, without any additional processing, is not suitable for the detection of SARS-CoV-2 via PCR.

Given the manifest disease in the lung as observed in CT scans, a positive SARS-CoV-2 swab in the upper respiratory tract without the lungs being affected is unlikely in patients needing invasive ventilation. Hence, the reason for the negative results remain unclear.

Unfortunately, the study did not control for prior inhalation therapy. Some of the fluid could have been nebulized saline instead of precipitated moisture from the distal airspace carrying viral RNA. Even so, viral RNA should still be detectable under these conditions.

Further processing of the samples, including the extraction of more liquid via centrifugation of the HME filters, might yield better results, though processing steps would render the technique less applicable and appealing for widespread use [5].

## Data Availability

Data can be made available upon request.
